# Supplementary figures and images for: A genome assembly and annotation for the Australian alpine skink Bassiana duperreyi using long-read technologies
Source: G3 (Bethesda). 2025 Mar 14;15(6):jkaf046. doi: 10.1093/g3journal/jkaf046 (PMC12134992; doi:10.1093/g3journal/jkaf046)

Average Read QV

ONT

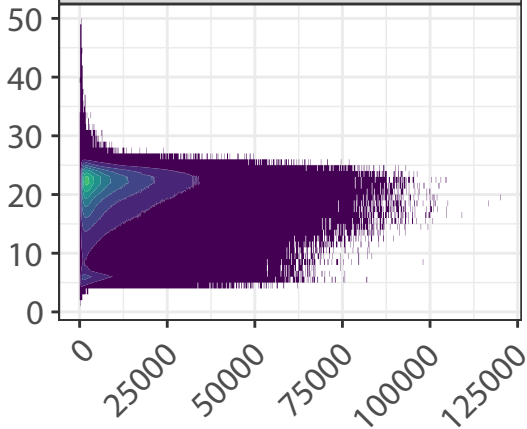

PacBio HiFi

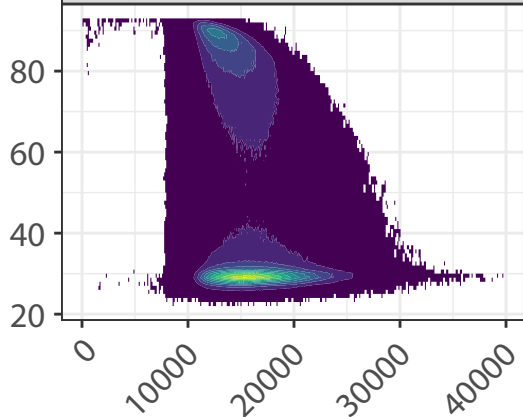

Read Length (bp)

Base Fraction

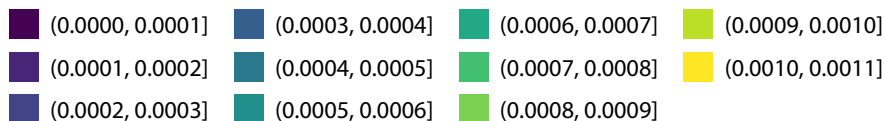

Supplement: jkaf046_Supplementary_Data [file jkaf046_supplementary_data.zip › Figure_S1_G3-2024-405360.pdf]

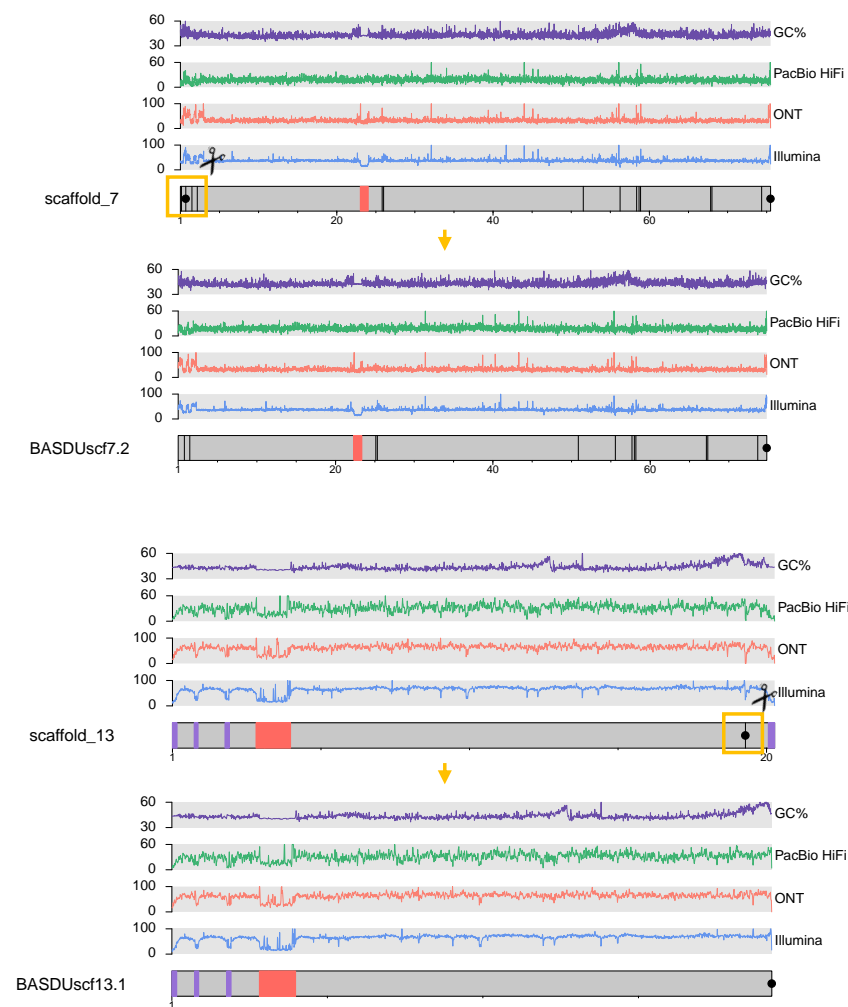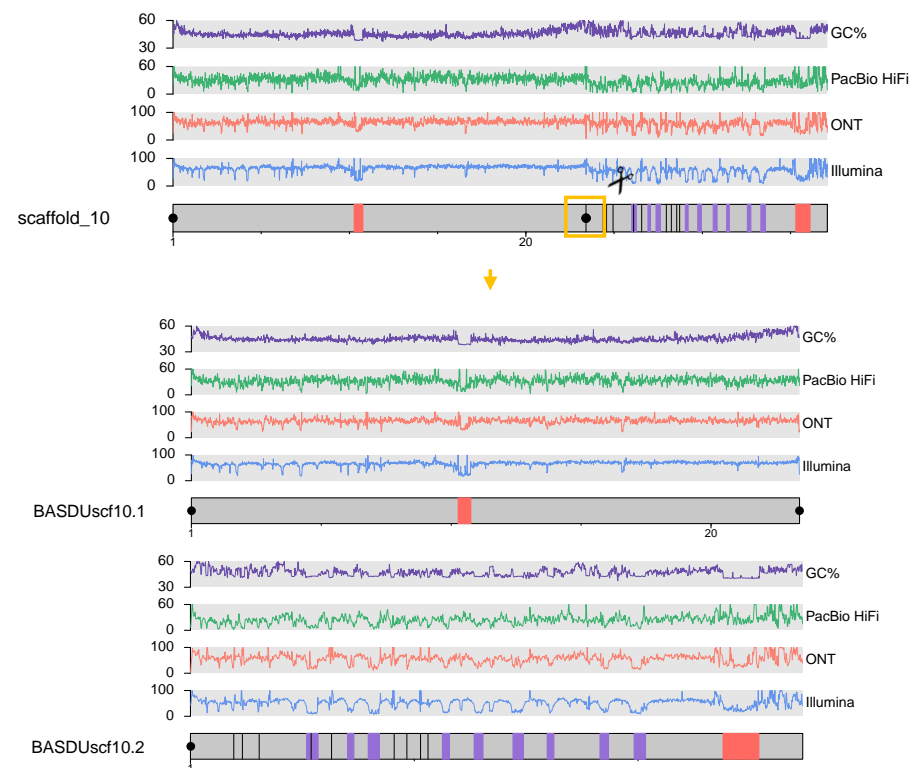

Supplement: jkaf046_Supplementary_Data [file jkaf046_supplementary_data.zip › Figure_S2_G3-2024-405360.pdf]

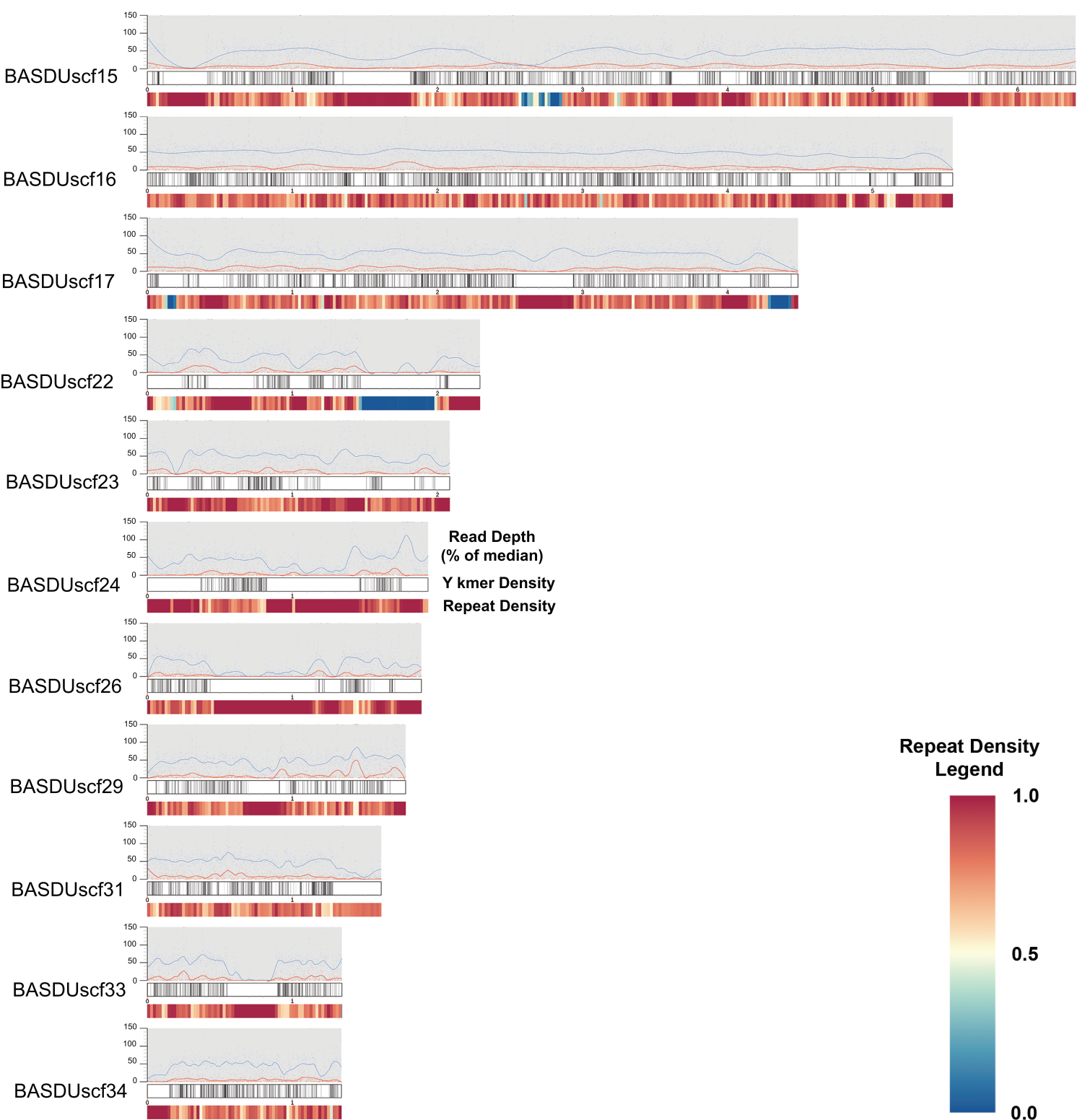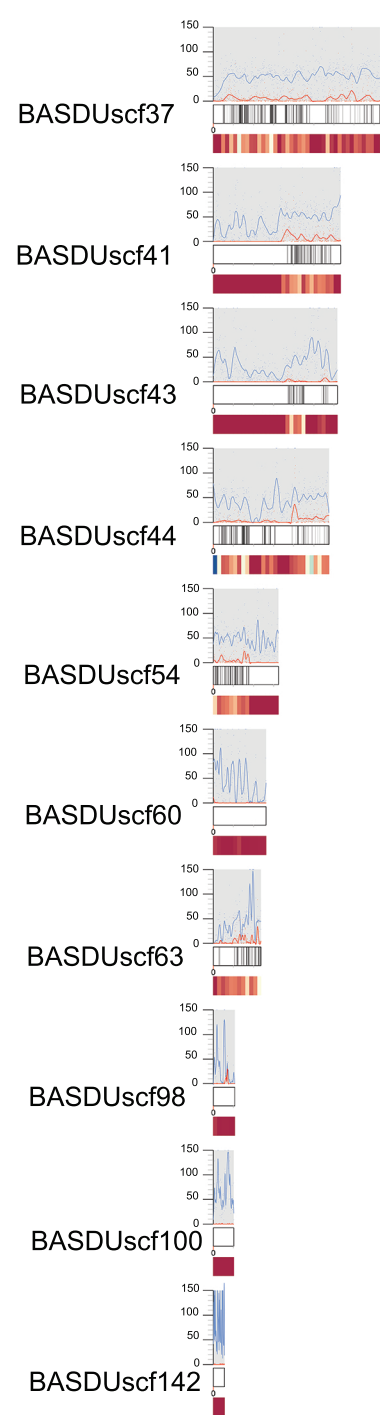

**Repeat Density  
Legend**

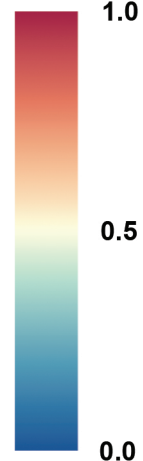

Supplement: jkaf046_Supplementary_Data [file jkaf046_supplementary_data.zip › Figure_S3_G3-2024-405360.pdf]

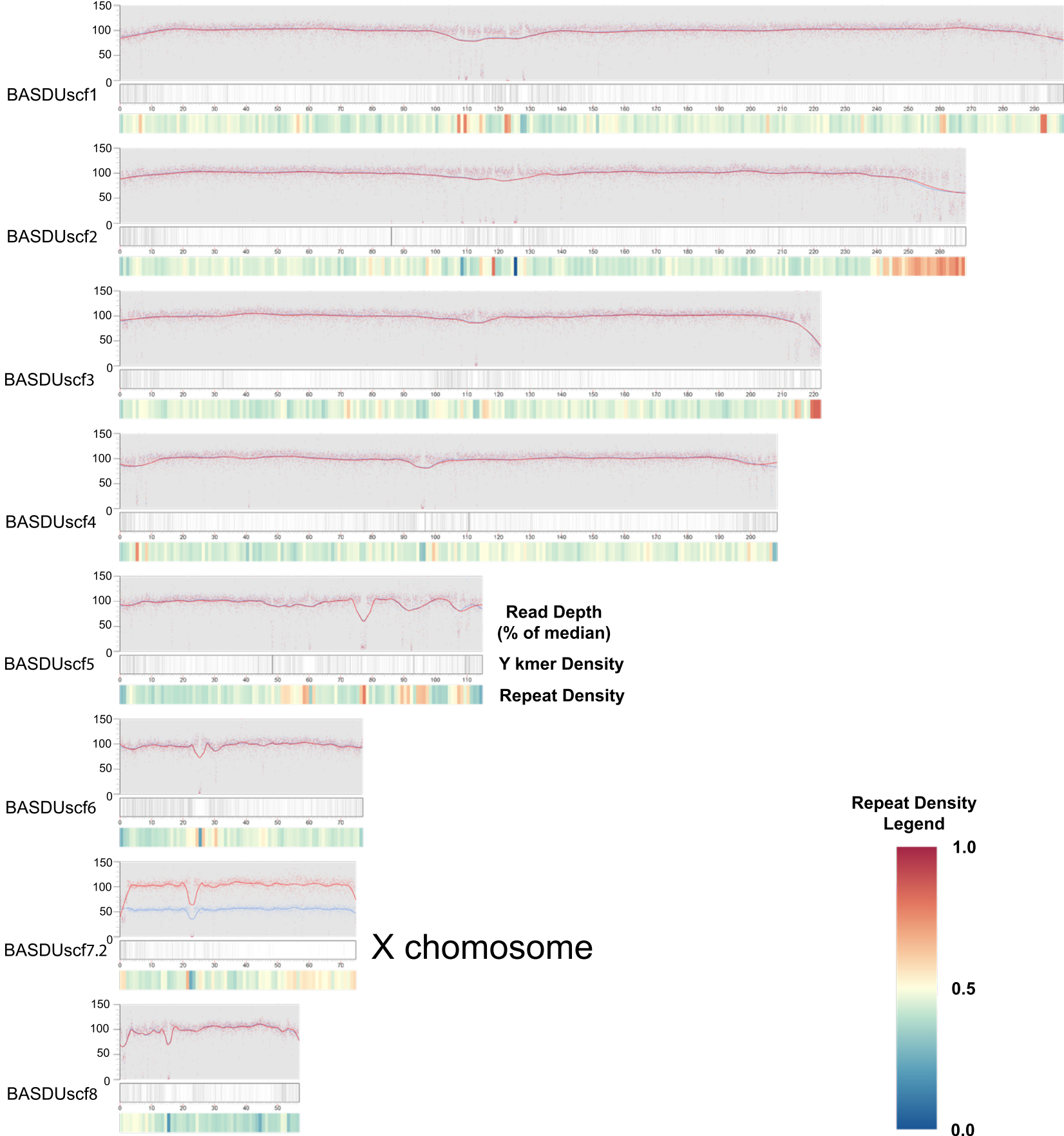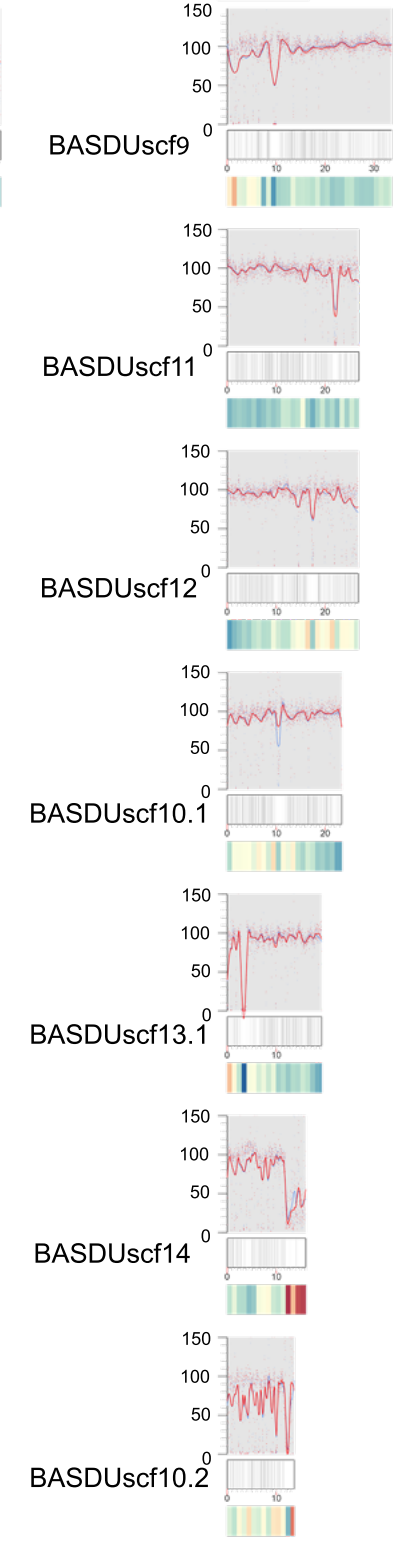

Supplement: jkaf046_Supplementary_Data [file jkaf046_supplementary_data.zip › Figure_S4_G3-2024-405360.pdf]

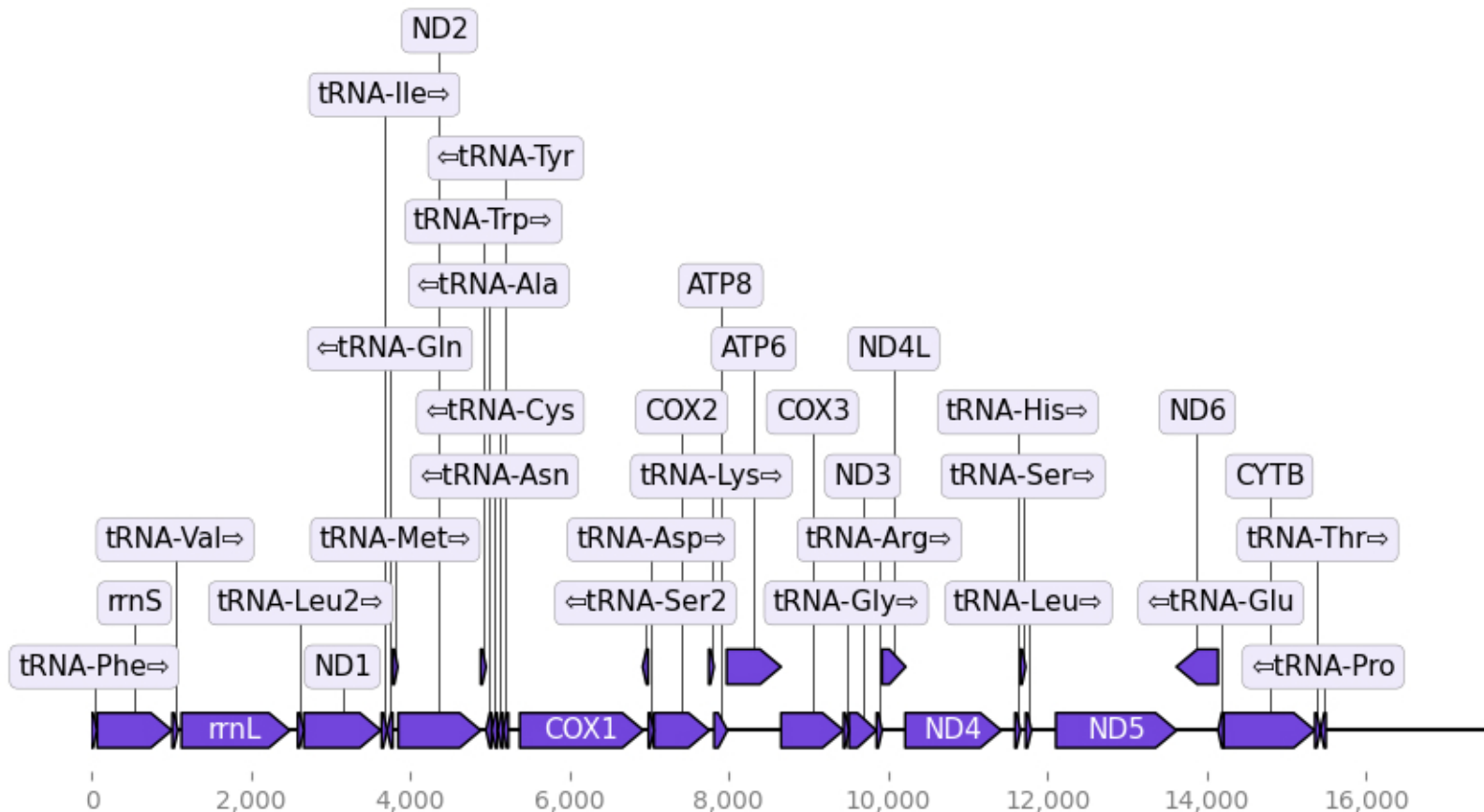

Supplement: jkaf046_Supplementary_Data [file jkaf046_supplementary_data.zip › Figure_S5_G3-2024-405360.pdf]

0

500

1000

1500

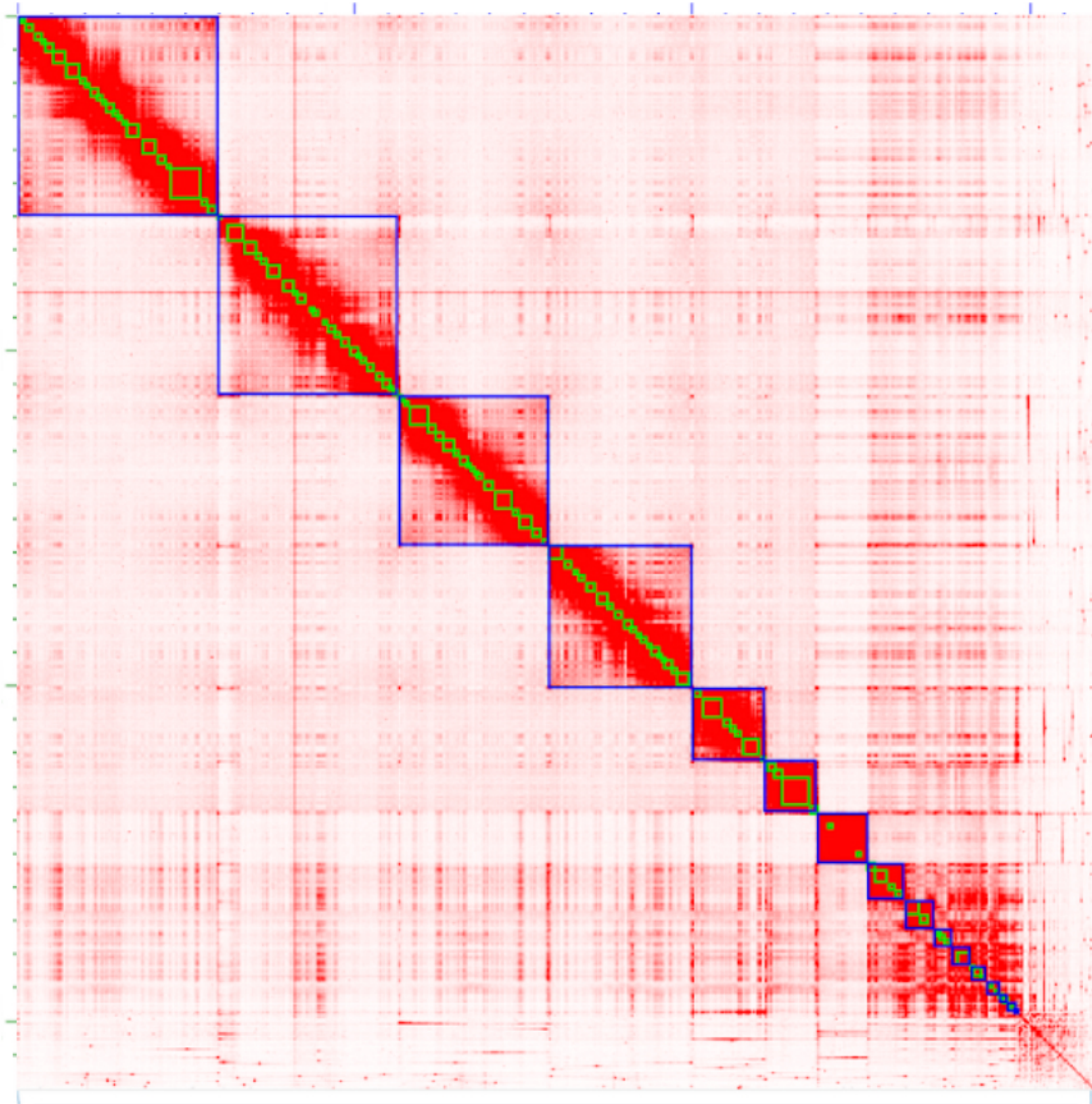

Supplement: jkaf046_Supplementary_Data [file jkaf046_supplementary_data.zip › Figure_S6_G3-2024-405360.pdf]
